# *Notes from the Field:* Outbreak of Ebola Virus Disease Caused by *Sudan ebolavirus* — Uganda, August–October 2022

**DOI:** 10.15585/mmwr.mm7145a5

**Published:** 2022-11-11

**Authors:** Thomas Kiggundu, Alex R. Ario, Daniel Kadobera, Benon Kwesiga, Richard Migisha, Issa Makumbi, Daniel Eurien, Zainah Kabami, Joshua Kayiwa, Bernard Lubwama, Denis Okethwangu, Susan Nabadda, Godfrey Bwire, Sophia Mulei, Julie R. Harris, Emilio Dirlikov, Arthur G. Fitzmaurice, Sandra Nabatanzi, Yonas Tegegn, Allan N. Muruta, Daniel Kyabayinze, Amy L. Boore, Atek Kagirita, Henry Kyobe-Bosa, Henry G. Mwebesa, Diana Atwine, Jane R. Aceng Ocero, Uganda Ebola Response Team, Uganda Ebola Response Team, Natalie E. Brown, Brian Agaba, Sherry Rita Ahirirwe, Rebecca Akunzirwe, Alice Asio, Immaculate Atuhaire, Sarah Elayeete, Doreen Gonahasa, Edirisa Juniour Nsubuga, Patrick King, Saudah Kizito, Allan Komackech, Veronica Masanja, Stella Martha Migamba, Patience Mwine, Hellen Nelly Naiga, Petranilla Nakamya, Rose Nampeera, Mackline Ninsiima, Alex Ndybakira, Lawrence Oonyu, Brenda Nakafeero Simbwa, Hildah Tendo, Mercy Wendy Wanyana, Jane Frances Zalwango, Maria Gorette Zalwango, Robert Zavuga, Immaculate Asiimwe, Richardson Mafigiri, Esther Muwanguzi, Stephen Balinandi, Luke Nyakarahuka, Jimmy Baluku, Jackson Kyando, Alex Tumusiime, Caitlin Cossaboom, Carrie Eggers, John D. Klena, Lisa Nelson, Modupe Osinubi, Katrin Sadigh, Waverly Vosburgh, Mary-Claire Worrell, Philimon Kabagambe, Charles Okot, Godfrey Nsereko, Olivia Namusisi

**Affiliations:** ^1^Uganda Public Health Fellowship Program, Uganda National Institute of Public Health, Kampala, Uganda; ^2^National Public Health Emergency Operations Centre, Uganda National Institute of Public Health, Kampala, Uganda; ^3^Baylor College of Medicine Children’s Foundation-Uganda, Kampala, Uganda; ^4^Uganda Ministry of Health, Kampala, Uganda; ^5^National Health Laboratory Services, Uganda Ministry of Health, Kampala, Uganda; ^6^Uganda Virus Research Institute, Kampala, Uganda; ^7^CDC Uganda, Kampala, Uganda; ^8^World Health Organization Country Office, Kampala, Uganda.; U.S. Department of State; Uganda National Institute of Public Health; Uganda National Institute of Public Health; Uganda National Institute of Public Health; Uganda National Institute of Public Health; Uganda National Institute of Public Health; Uganda National Institute of Public Health; Uganda National Institute of Public Health; Uganda National Institute of Public Health; Uganda National Institute of Public Health; Uganda National Institute of Public Health; Uganda National Institute of Public Health; Uganda National Institute of Public Health; Uganda National Institute of Public Health; Uganda National Institute of Public Health; Uganda National Institute of Public Health; Uganda National Institute of Public Health; Uganda National Institute of Public Health; Uganda National Institute of Public Health; Uganda National Institute of Public Health; Uganda National Institute of Public Health; Uganda National Institute of Public Health; Uganda National Institute of Public Health; Uganda National Institute of Public Health; Uganda National Institute of Public Health; Uganda National Institute of Public Health; Uganda National Institute of Public Health; Uganda Ministry of Health; Uganda Ministry of Health; Uganda Ministry of Health; Uganda Virus Research Institute; Uganda Virus Research Institute; Uganda Virus Research Institute; Uganda Virus Research Institute; Uganda Virus Research Institute; CDC; CDC; CDC; CDC; CDC; CDC; CDC; CDC; World Health Organization; Uganda; World Health Organization, Uganda; CDC; Africa Field Epidemiology Network

Ebola virus disease (EVD) is a rare and often deadly viral hemorrhagic fever (VHF); four species of Ebola virus (*Zaire ebolavirus*,* Sudan ebolavirus*,* Taï Forest ebolavirus*, and* Bundibugyo ebolavirus*) cause occasional outbreaks among humans and nonhuman primates* (*1*). Infection is transmitted through direct contact with infectious blood, body fluids, and animal tissues. Symptoms include fever, abdominal pain, diarrhea, vomiting, generalized body weakness, and hemorrhage. Since 2000, four outbreaks of EVD caused by *Sudan ebolavirus* have been identified in Uganda; the largest outbreak (in 2000) resulted in 425 cases and 224 (53%) deaths (*2*,*3*). No vaccine is available to prevent *Sudan ebolavirus* infection, and treatment is supportive. The estimated case fatality rate is 55% (*4*).

On September 17, 2022, Uganda’s National Public Health Emergency Operations Centre and the Uganda Virus Research Institute VHF program were notified of a suspected VHF case in a male patient (patient A) aged 26 years who lived in Madudu subcounty, Mubende District in central Uganda. The patient had been referred to Mubende Regional Referral Hospital (MRRH) the previous day by a private health clinic. At the time of admission, patient A had high fever, abdominal pain, diarrhea, chest pain, loss of appetite, dry cough, bloody vomitus, and bleeding from the eyes. He reported no recent travel or known Ebola virus exposures. Based on public health protocols for suspected VHF, the patient was isolated at MRRH by the Mubende District health team to await laboratory test results. Despite supportive treatment, the patient died on September 18, 2022, and the Uganda Ministry of Health (MoH) was notified. A blood sample collected from patient A on September 17 was confirmed positive for *Sudan ebolavirus* on September 19; testing by reverse transcription–polymerase chain reaction (RT-PCR) had been conducted at the Uganda Virus Research Institute (*5*). On September 19, local leaders from Madudu and Butoloogo, two contiguous subcounties, reported six community deaths during August 13**–**September 15 among persons with probable EVD. Upon laboratory confirmation of EVD in patient A, MRRH established an Ebola treatment unit (ETU) to manage patients, and another ETU was activated at Fort Portal Regional Referral Hospital in western Uganda.

On September 21, 2022, MoH, the Mubende District health team, and other partners launched a public health emergency response to identify additional cases of EVD. The response focuses on active case finding (initiated in Madudu and Butoloogo subcounties), tracing and monitoring contacts for EVD signs and symptoms, conducting laboratory testing, and providing treatment. A probable case was defined as death in a person with suspected EVD who had a direct epidemiologic link to a confirmed case but did not have laboratory-confirmed infection. A confirmed case was a person with EVD who had received positive laboratory test results by RT-PCR or EVD immunoglobulin M serology. Patients with EVD who were discharged from ETUs with two negative RT-PCR tests 48 hours apart were considered recovered. Contact monitoring consisted of daily temperature checks and EVD symptom screening of persons who had direct (i.e., physical) or indirect contact (e.g., with used linens or utensils, or shared space) with patients with probable or confirmed EVD.

During September 18–October 31, 2022, a total of 130 confirmed and 18 probable EVD cases were identified; symptom onset in the first probable case occurred on August 8 (Figure). On October 31, a total of 1,777 contacts were monitored; among these 1,540 (87%) were successfully monitored on that day. Among probable and confirmed cases, the median patient age is 29 years (range = 1–70 years), 87 (59%) patients are men, and 81 (55%) are residents of Mubende district. Among the 130 confirmed cases, 43 (33%) deaths were reported as of October 31. Sixty-one deaths (probable and confirmed cases) were reported; the median age of decedents was 28 years (range = <1–58 years), and 31 (51%) were women. Eighteen confirmed or probable EVD cases during this period were reported among health care workers; five were linked to a surgical procedure performed on a patient with probable EVD who died on September 17. As of October 31, 45 patients had recovered, 37 patients are undergoing treatment in an ETU, and outcomes are unknown for five.

**FIGURE:**
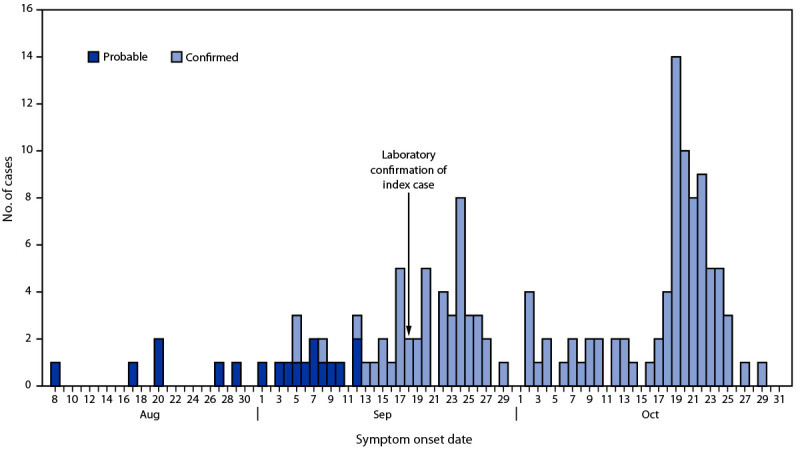
Probable (n = 18) and confirmed (n = 130) cases of Ebola virus disease,* by symptom onset date^†^ — Uganda, August 8–October 31, 2022 **Abbreviations:** EVD = Ebola virus disease; RT-PCR = reverse transcription–polymerase chain reaction. * A probable case was defined as death in a person with suspected EVD who had a direct epidemiologic link to a confirmed case but did not have laboratory-confirmed infection. A confirmed case was a person with EVD who had received positive laboratory test results by RT-PCR or EVD immunoglobulin M serology. ^†^ Identified cases as of October 31, 2022. Additional cases with symptom onset on or before October 31, might subsequently be identified.

This report describes the fifth outbreak of EVD caused by *Sudan ebolavirus* in Uganda since 2000 (*2*,*3*). Given continued transmission, public health response to this outbreak is ongoing, including epidemiologic investigation to identify the source of the outbreak.^†^ Persons living in or traveling to Uganda should avoid activities that could result in exposures to infected persons or animals, including direct contact with body fluids or objects contaminated with blood or body fluids. Health care workers caring for EVD patients should adhere to proper infection control practices, including appropriate use of personal protective equipment.^§^ Public health departments, public health laboratories, and health care workers outside Uganda, including in the United States, should be aware of recommended practices to identify, report, and prevent EVD.^¶^ Health care providers in the United States and elsewhere should be alert for and evaluate any patients suspected of having EVD, particularly persons who have recently been in the affected areas in Uganda.

## Footnotes

*
https://www.cdc.gov/vhf/ebola/index.html

†
https://www.cdc.gov/vhf/ebola/prevention/index.html

§
https://www.cdc.gov/niosh/topics/ebola/longdev/healthcare.html

¶ https://emergency.cdc.gov/han/2022/han00480.asp (Accessed November 7, 2022)
